# Supplementary material for: Poly(9H-carbazole) as a Organic Semiconductor for Enzymatic and Non-Enzymatic Glucose Sensors
Source: Biosensors (Basel). 2020 Aug 23;10(9):104. doi: 10.3390/bios10090104 (PMC7560144; doi:10.3390/bios10090104)
Supplement: Supplementary file 1 [file biosensors-10-00104-s001.pdf]

biosensors

Supporting Information for Poly(*9H*-carbazole) as a Organic Semiconductor for Enzymatic and Non-Enzymatic Glucose Sensors

Gintautas Bagdžiūnas[1,2]* Delianas Palinauskas[1]

[1] Institute of Biochemistry, Life Sciences Centre, Vilnius University, Sauletekio av. 7, LT- 10257, Vilnius, Lithuania

[2] Department of Functional Materials and Electronics, Center for Physical Sciences and Technology, Sauletekio av. 3, LT-10257 Vilnius, Lithuania

* Correspondence: G. Bagdziunas (ORCID ID: orcid.org/0000-0002-9924-6902), E-mail: gintautas.bagdziunas@gmc.vu.lt

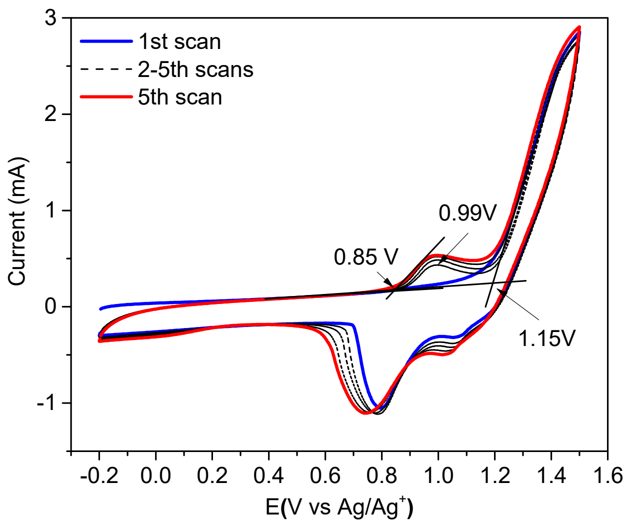

Figure 1. Electrochemical synthesis of polyCz from *9H*-carbazole as the monomer in dry dichloromethane solution with $TBAPF_6$ (0.1 M) as the supporting electrolyte at sweep rate of 50 mV s^{-1}.

Table 1. Calculated pseudocapacitive and faradaic current densities for the polyCz and polyCz/GOx electrodes at -0.50, 0.35 and 0.80 V vs Ag/AgCl.

Current densities in mA/cm2										
	pseudocapacitive	faradaic	pseudocapacitive	faradaic	pseudocapacitive	faradaic	pseudocapacitive	faradaic	pseudocapacitiv	faradaic
	PolyCz						PolyCz/GOx			
Sveep rate, V/s	at -0.5 V		at 0.35 V		at 0.8 V		at -0.5 V		at 0.35 V	
0.0100	0.0057	-0.4682	0.0516	0.1423	-0.0020	0.3982	0.0339	-0.5755	0.0440	0.1945
0.0200	0.0113	-0.6621	0.1032	0.2012	-0.0040	0.5632	0.0678	-0.8138	0.0880	0.2751
0.0300	0.0170	-0.8109	0.1548	0.2464	-0.0061	0.6898	0.1016	-0.9967	0.1320	0.3369
0.0400	0.0226	-0.9364	0.2065	0.2846	-0.0081	0.7965	0.1355	-1.1509	0.1760	0.3891
0.0500	0.0283	-1.0469	0.2581	0.3181	-0.0101	0.8905	0.1694	-1.2868	0.2201	0.4350
0.0600	0.0339	-1.1468	0.3097	0.3485	-0.0121	0.9755	0.2033	-1.4096	0.2641	0.4765
0.0700	0.0396	-1.2387	0.3613	0.3764	-0.0141	1.0536	0.2372	-1.5225	0.3081	0.5147
0.0800	0.0453	-1.3242	0.4129	0.4024	-0.0161	1.1264	0.2711	-1.6276	0.3521	0.5502
0.0900	0.0509	-1.4046	0.4645	0.4268	-0.0182	1.1947	0.3049	-1.7264	0.3961	0.5836
0.1000	0.0566	-1.4805	0.5161	0.4499	-0.0202	1.2593	0.3388	-1.8197	0.4401	0.6152

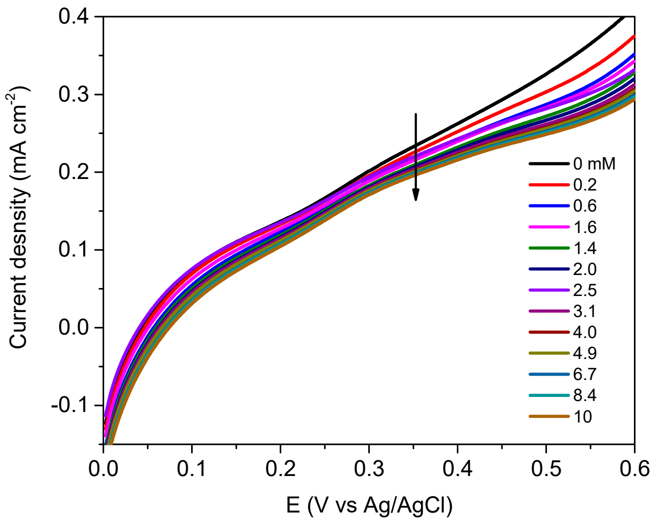

Figure 2. Current density responses for glucose detection on the enzymatic polyCz-Fe/GOx electrode by using differential pulse voltammetry (DPV) from 0 to 0.6 V vs Ag/AgCl.
